# Outcomes of robot-assisted laparoscopic extended pelvic lymph node dissection for prostate Cancer

**DOI:** 10.1186/s12894-024-01409-8

**Published:** 2024-01-29

**Authors:** Silvan Sigg, Fabienne Lehner, Etienne Xavier Keller, Karim Saba, Holger Moch, Tullio Sulser, Daniel Eberli, Ashkan Mortezavi

**Affiliations:** 1 Department of Urology, University Hospital Zurich, University of Zurich, Zurich, Switzerland; 2 Urologiezentrum Hirslanden, Hirslanden Klinik Aarau, Aarau, Switzerland; 3 Department of Pathology and Molecular Pathology, University Hospital Zurich, Zurich, Switzerland; 4 Department of Urology, University Hospital Basel, University of Basel, Basel, Switzerland; 5 Department of Medical Epidemiology and Biostatistics, Karolinska Institutet, Stockholm, Sweden

**Keywords:** Outcome, Lymph node dissection, Prostate cancer, Laparoscopic, Robotic, da Vinci

## Abstract

**Introduction:**

Extended pelvic lymph node dissection (ePLND) in men undergoing robot-assisted laparoscopic radical prostatectomy (RARP) is a widely used procedure. However, little is known about anatomical site-specific yields and subsequent metastatic patterns in these patients.

**Patients and methods:**

Data on a consecutive series of 1107 patients undergoing RARP at our centre between 2004 and 2018 were analysed. In men undergoing LN dissection, the internal, external and obturator nodes were removed and sent in separately. We performed an analysis of LN yields in total and for each anatomical zone, patterns of LN metastases and complications. Oncological outcome in pN+ disease was assessed including postoperative PSA persistence and survival.

**Results:**

A total of 823 ePLNDs were performed in the investigated cohort resulting in 98 men being diagnosed as pN+ (8.9%). The median (IQR) LN yield was 19 (14–25), 10 (7–13) on the right and 9 (6–12) on the left side (*P* < 0.001). A median of six (4–8) LNs were retrieved from the external, three (1–6) from the internal iliac artery, and eight (6–12) from the obturator fossa. More men had metastatic LNs on the right side compared to the left (41 vs. 19). Symptomatic lymphoceles occurred exclusively in the ePLND group (2.3% vs. 0%, *p* = 0.04). Postoperatively, 47 (47.9%) of men with pN+ reached a PSA of < 0.1μg/ml. There was no association between a certain pN+ region and postoperative PSA persistence or BCRFS. The estimated cancer specific survival rate at 5 years was 98.5% for pN+ disease.

**Conclusion:**

Robot-assisted laparoscopic ePLND with a high LN yield and low complication rate is feasible. However, we observed an imbalance in more removed and positive LNs on the right side compared to the left. A high rate of postoperative PSA persistence and early recurrence in pN+ patients might indicate a possibly limited therapeutical value of the procedure in already spread disease. Yet, these men demonstrated an excellent survival.

**Supplementary Information:**

The online version contains supplementary material available at 10.1186/s12894-024-01409-8.

## Introduction

Prostate cancer (PCa) disseminates initially and primarily to regional lymph nodes (LNs). This observation has caused pelvic LN dissection (PLND) to become a standard procedure during radical prostatectomy (RP). The European (EAU) and American (AUA) urological guidelines endorse usage of nomograms for patient selection and an anatomically extended template (ePLND) [1–4].

Being part of the same procedure, analogue to prostatectomy a laparoscopic robot-assisted approach for removal of the nodes has become the standard approach. Opposing early concerns of a lower LN count with the robotic approach, several studies have shown a comparable yield [5, 6]. However, it remains unclear if due to the technical set up of the robotic arms and their working angles all anatomical regions can be achieved equally well.

Regardless of the surgical approach, additional performance of PLND is often associated with worse intraoperative and perioperative outcomes [7, 8]. This gains significance considering the uncertain therapeutical benefit of PLND [1, 9, 10]. It has been claimed that if the involved regional nodes represent the only site of systemic disease, appropriate surgical extirpation may be able to cure the patient and decrease the cancer specific mortality [11]. However, hard scientific evidence proving this concept, especially in prospective trials, is still missing. Others have proposed the diagnostic value of PLND as a justification for the procedure since nodal positive men with PCa undergoing RP may benefit from early adjuvant treatment [12]. However, the true rate of patients and urologists opting for early adjuvant androgen deprivation therapy (ADT) or adjuvant external beam radiation therapy (EBRT) remains unknown [13] and the diagnostic value becomes more arguable in the era of PSMA-PET/CT [14].

In order to contribute additional data and shed more light into an area of urologic oncology with low evidence level, we performed an in-depth analysis of outcomes in a large cohort of men undergoing robot-assisted ePLND for PCa. Our primary aim was to assess the LN yield stratified for side and anatomical region. Secondarily, we aimed to study metastatic patterns, complication rates and the potential therapeutic benefit in removal of regional metastatic LNs in individuals with node-positive disease.

## Patients and methods

### Patients and data acquisition

We retrospectively collected clinicopathological data of a consecutive series of men with localized PCa who underwent robotic-assisted RP (RARP) at a single centre between 04/2004 and 09/2017. Excluded were patients who previously received ADT or EBRT. Covariates included age, body mass index (BMI), preoperative PSA and haemoglobin value, clinical T-stage, biopsy Gleason score, and performance of nerve-sparing. Patients were stratified by D’Amico risk categories [15]. The local ethics committee approved the present study (KEK-ZH-Nr. 2017–02335).

### Surgical technique

A transperitoneal Da-Vinci-robot-assisted approach was used in all patients as previously described [5]. All procedures were performed with a grasping instrument placed on the left and scissors or a hook on the right. In general, indication for PLND was a Gleason score ≥ 7 or PSA level ≥ 10 ng/ml. However, the decision was not based on any risk score but on surgeons and patients’ discretion. Routinely, an ePLND was performed with boundaries according to Bader et al. [16]. ePLND started with removing the LNs nodes covering the external iliac vessels between the ureter (upper) and the deep circumflex iliac vein (lower boundary). The obturator fossa LNs were removed after identification of the obturator nerve beneath the external iliac vein and the pelvic wall. Finally, the lymphatic tissue along the internal iliac artery with the obliterated umbilical artery as medial boundary were removed. LNs from each region were retrieved and sent to the pathologist separately. Histopathological evaluation of lymphadenectomy specimens was performed according to recommendations in pathology [17]. In detail, during the entire study period large-format histology was employed with the total submission of the dissected tissues without any frozen sections.

### Outcomes and oncological follow-up

Postoperative parameters included intraoperative complications, haemoglobin on the first postoperative day, surgical margin status, pathological stage, final Gleason score, number of removed LNs (per region) and nodal status. Haemoglobin difference was defined as the change between the preoperative and postoperative value. We evaluated the occurrence of symptomatic lymphoceles, transfusions and reoperations within 90 days after surgery and categorized them using the modified Clavien-Dindo classification [18]. Follow-up visits occurred 6 weeks, 3, 6, and 12 months after RALRP, and then yearly with physical examination and PSA testing. The referring urologists or the patients’ general practitioners provided outcome data if the follow-up examinations were not performed at our centre. The outcome measures were biochemical recurrence-free survival (BCRFS), overall (OS) and cancer-specific survival (CSS). Biochemical recurrence (BCR) was defined as a PSA value ≥0.1 ng/mL with subsequent confirmation after reaching the PSA nadir of 0.1 ng/ml postoperatively. Patients not reaching this nadir threshold postoperatively were excluded from BCRFS analysis. Any reported death in the records was registered and assessed whether it was PCa related. According to internal guidelines adjuvant EBRT, immediate ADT as well as observation were offered to nodal positive (pN+) patients. In cases of non-measurable postoperative PSA observation and deferred treatment was favoured over an immediate approach. The timing and the type of postoperative treatment was evaluated for all pN+ patients.

### Statistical analysis

We compared perioperative parameters of pN+ and pN- patients using the Pearson chi-square for categorial and Mann-Whitney-U test for continuous variables, respectively. Categorical endpoints were assessed using logistic regression. Univariate cox-regression analysis was performed to evaluate the association of the clinicopathological parameters on survival. BCRFS, OS and CSS curves were calculated using the Kaplan–Meier method with significance evaluated by 2-sided log-rank statistics. Calculations were performed using IBM SPSS Version 25 [19]. A *p*-value < 0.05 was considered as statistically significant.

## Results

### Patient cohort

Between 2004 and 2017 we performed a total number of 1134 RARPs at our centre, of which 27 patients were excluded from analysis due to preoperative ADT. Of the remaining 1107 patients, 827 (75%) underwent ePLND. A total of 11 surgeons were involved with 5 accounting for 92% of the radical prostatectomies. For baseline characteristics see Table 1. In total, 16`404 LN were removed, of which 256 (1.5%) were positive. Among patients undergoing ePLND, 98 (12%) patients were classified as pN+ on final pathology (8.9% of the total RARP cohort). These men had significantly higher preoperative PSA values, clinical T-stages and biopsy Gleason scores (*p* < 0.001); only 2% were classified as low-risk preoperatively.

**Table 1:** Baseline characteristics and perioperative parameters

	No PLND (*n* = 280)	pN- (*n* = 729)	pN+ (*n* = 98)	*p*-value (pN- vs. pN+)
**Preoperative data**				
**Age (years)**	62 (57–67)	64 (59–68)	63 (60–68)	0.935
**Body mass index (kg/m**^**2**^**)**	25.9 (23.4–28)	26.1 (23.9–28.7)	26.0 (23.9–28.6)	0.688
Missing	5 (1.7%)	5 (0.7%)	1 (0.1%)	
**Preoperative PSA (ng/ml)**	5.9 (4.5–7.9)	7.5 (5–12)	13.4 (8.5–22.8)	< 0.001
Missing	0 (0%)	0 (0%)	0 (0%)	
**Clinical Stage**				< 0.001
T1c	238 (85%)	594 (81.6%)	61 (62.2%)	
T2	42 (15%)	126 (17.3%)	27 (27.6%)	
T3	0 (0%)	8 (1.1%)	10 (10.2%)	
Missing	0 (0%)	0 (0%)	0 (0%)	
**Biopsy Gleason Score**				< 0.001
6	248 (88.6%)	119 (16.3%)	9 (9.2%)	
7a	22 (7.9%)	325 (44.6%)	27 (27.6%)	
7b	9 (3.2%)	141 (19.3%)	19 (19.4%)	
8	0 (0%)	83 (11.4%)	17 (17.3%)	
9–10	0 (0%)	54 (7.5%)	24 (24.4%)	
Missing	1 (0.4%)	7 (1.0%)	2 (2.0%)	
**D’Amico Risk Group**				< 0.001
Low	196 (70%)	63 (8.6%)	2 (2%)	
Intermediate	67 (23.9%)	450 (61.7%)	26 (26.5%)	
High	17 (6.1%)	216 (29.6%)	70 (71.4%)	
Missing	0 (0%)	0 (0%)	0 (0%)	
**Perioperative data**				
**Nerve sparing, any**	237 (85.3%)	447 (61%)	30 (31.9%)	< 0.001
Missing	2 (0.7%)	3 (0.4%)	4 (4%)	
**Hemoglobin difference (g/l)**	3.4 (2.6–4.3)	2.9 (2.3–3.7)	2.8 (2.1–3.8)	0.756
Missing	18 (6.4%)	41 (5.6%)	2 (2%)	
**Pathology results**				
**Positive surgical margins**	76 (27.1%)	209 (28.7%)	53 (54.1%)	< 0.001
**pT Stage**				< 0.001
pT2a-T2c	249 (88.9%)	499 (68.4%)	9 (9.2%)	
pT3a	23 (8.2%)	176 (24.1%)	41 (41.8%)	
pT3b-pT4^a^	6 (2.1%)	50 (6.9%)	44 (44.9%)	
Missing	2 (0.7%)	4 (0.5%)	4 (4.1%)	
**Gleason Score**				< 0.001
6	117 (41.5%)	47 (6.4%)	0 (0%)	
7a	114 (41%)	332 (45.6%)	12 (12.2%)	
7b	37 (13.2%)	195 (26.7%)	20 (20.4%)	
8	8 (2.9%)	58 (8%)	22 (22.4%)	
9–10	2 (0.7%)	96 (13.1%)	39 (39.8%)	
Missing	2 (0.7%)	1 (0.1%)	5 (5.1%)	
**Number of resected LNs**	–	19 (13–24)	19 (15–27)	0.307

Data presented as median and inter quartile range (IQR) or as n (%).

*GS* Gleason score: *LN* lymph node: *PLND* pelvic lymph node dissection

^a^a total of four pT4 cases in the entire cohort

### Lymph node distribution

**Fig. 1:**
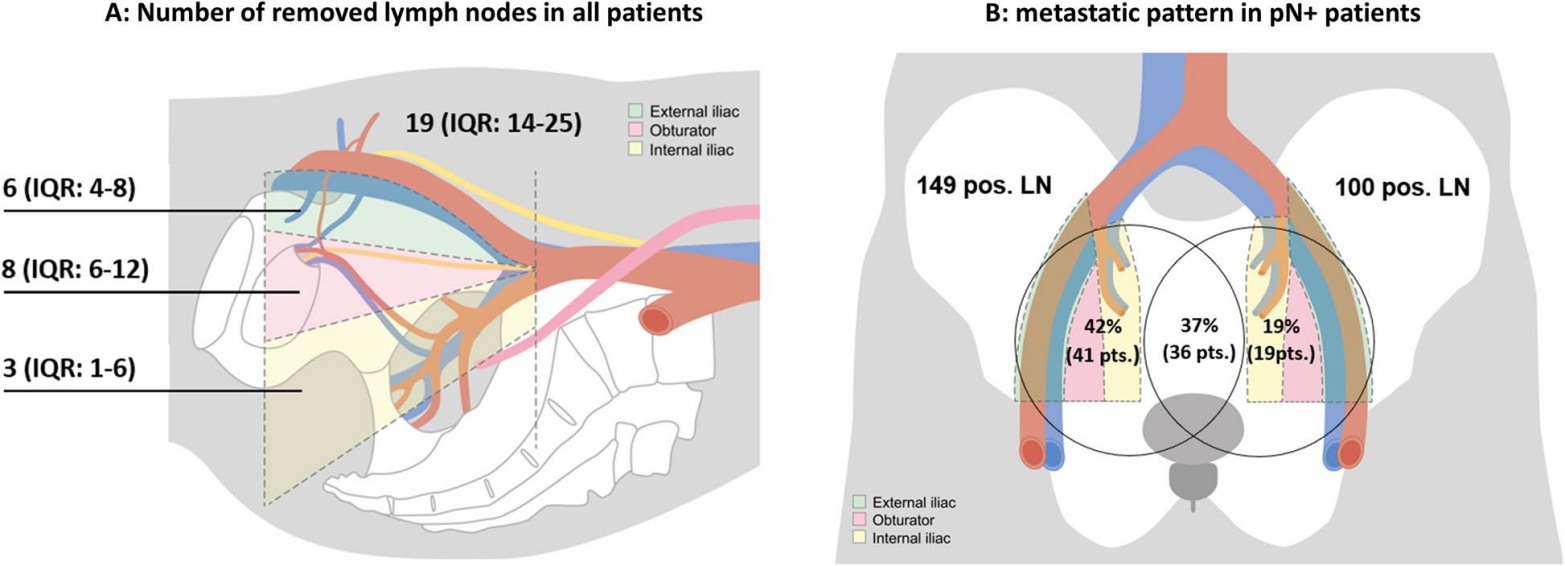
**A** Sagittal view on pelvis with visualization of the 3 zones of the extended pelvic lymph node dissection and the total number of removed lymph nodes. IQR: interquartile range. **B** Frontal view on pelvis displaying the number of positive lymph nodes on each side and the metastatic pattern per patient. Not shown: one patient with preprostatic metastasis, one patient with missing data, 4 metastatic periprostatic LNs. LN: lymph node, pts.: patients

A median number of 19 (interquartile range [IQR]: 14–25) LNs were removed, 10 (IQR: 7–13) on the right and 9 (IQR: 6–12) on the left side (p < 0.001). A median of six (IQR: 4–8) LNs were retrieved from the external iliac vessels, eight (IQR: 6–12) from the obturator fossa and three (IQR: 1–6) from the internal iliac arteries (Fig. 1A). There was a significantly higher number of removed total nodes per region when comparing sides in regards to the obturator (mean [standard deviation], 4.9 ± 3 on the right vs. 4.5 ± 3 on the left, *p* = 0.002) and the external (right: 3.2 ± 2 vs. left: 2.9 ± 2, *p* = 0.005), but not the internal (right: 2.1 ± 2 vs. left: 1.9 ± 2, *p* = 0.1) nodes.

### LN metastasis

**Fig. 2:**
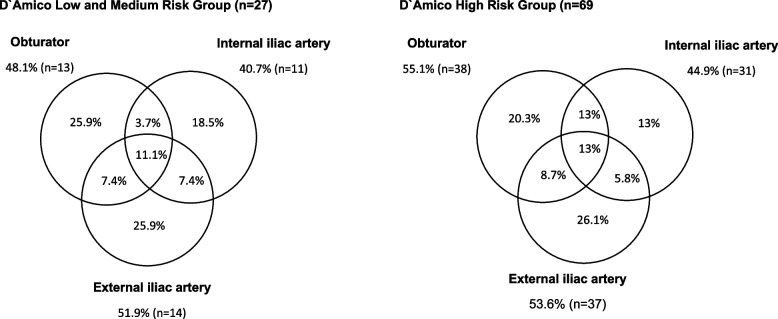
Distribution pattern of lymph node positive regions in A) D’Amico low and intermediate risk (*n* = 28) and B) in in D ‘Amico high risk patients (*n* = 70). Missing: 2, periprostatic: 2

The median number of positive LNs per patient was two (IQR 1–3); 46% (*n* = 45) had only one, 19% (*n* = 19) had two, and 35% (*n* = 34) had more than two positive LNs. Among the pN+ patients (*n* = 98), 49% (*n* = 48) had uni- or bilateral positive LNs around the external iliac artery, 52% (*n* = 51) in the obturator fossa and 43% (*n* = 42) in the region of the internal iliac artery. Four Patients had periprostatic positive lymph nodes (4.1%). Significantly more men had metastatic LNs on the right side compared to left (Fig. 1B). Patient classified as high risk had more often positive LNs in multiple regions than patients within the low or intermediate risk group (Fig. 2).

In the pN+ population, a total number of 2065 LNs were removed (1059 on the right, 976 on the left; missing: 30); 255 were positive (12%), 149 on the right (14.1%) and 100 on the left side (10.2%) (Fig. 1B; 4 periprostatic, missing: 2). The rate of positive nodes per region based on the total removed nodes in the corresponding area in the pN+ cohort was 14% (86/615) around the external iliac artery, 9.7% (88/909) in the obturator fossa and 18% (82/447) in the region of the internal iliac artery. For the entire ePLND cohort, these rates were 1.7, 1.1, and 2.5%, respectively.

The internal iliac nodes were positive in 43% (*n* = 42) of all pN+ patients. If the internal iliac nodes would not have been resected, 14 men (14.3%) would have been falsely staged as pN- and a total of 82 positive LNs (33%) would have been left in situ. Based on the total ePLND cohort, the number of men who would need to undergo a dissection with inclusion of the internal nodes to detect one additional pN+ patient was 59.

### Complications

Symptomatic lymphoceles occurred in 2.1% of all patients with ePLND, whereas none were found in men without (*p* = 0.006, Supplementary Table 1). Clavien Dindo Grade IIIA and Grade IIIB complications occurred in 1.6 and 3.3% of all ePLND patients, compared to 0.3 and 1.1% in patients without PLND (OR 3.49, CI 1.24–9.84, *p* = 0.012). We observed increasing rates of complications (≥ Clavien Dindo Grade IIIA) over time among patients who underwent ePLND (2004–2008: 4.2%, 2009–2012 2.4%, 2013–2017 7.3%, overall 4.8%).

Significant bleeding requiring transfusions occurred in 2.1% of all ePLND patients vs. 4.7% in men not undergoing the procedure (OR 0.43, 95% CI 0.21–0.90, *p* = 0.024). The mean haemoglobin difference (g/l) pre- and postoperatively between patients with lymph node dissection (3.1 g/l, IQR 2.2–3.7) was not significantly smaller compared to patients without the procedure (3.6 g/l, IQR 2.6–4.3, *p* < 0.001).

### Oncological outcome of N+ patients

Postoperatively, 47 (47.9%) of men with nodal metastases reached the PSA nadir of < 0.1μg/ml and 50 (51%) experienced a PSA persistence (lost to follow-up: 1). For men with 1, 2 or more than two positive LNs the rate of PSA persistence was 38.6, 52.4 and 66.7%, respectively. There was no significant association between a certain N+ region (internal, external, obturator or multiple) and postoperative PSA persistence or the BCRFS rate (Table 2). Univariate logistic regression revealed that LN metastasis (OR 17.7, 95% CI: 10.6–27.7; p < 0.001) and seminal vesicle invasion (OR 17.2, 95% CI: 9.8–30.3, p < 0.001) were the strongest predictors for postoperative PSA persistence. Other significant predictors were a higher PSA value, extraprostatic tumour extension, positive surgical margin, and a Gleason score of 8–10 (Supplementary Table 2A).

**Table 2:** Clinicopathological characteristics and outcome of men with pN+ classified for different anatomical regions

	External	Internal	Obturator	Ext. + Int.	Ext. + Obt.	Int. + Obt.	Ext. + Int. + Obt.
**Patients (n)**	23	14	21	6	8	10	12
**Baseline**							
PSA (median (IQR)) (ng/ml)	18 (9–36)	16 (11–32)	12 (6–26)	19.7 (15–45)	7.3 (4–39)	56.9 (14–91)	21.8 (20–40)
**Histopathology (prostatectomy)**							
GS 6	–	–	–	–	–	–	–
GS 7a	2 (9%)	1 (7%)	6 (29%)	–	–	2 (20%)	1 (8%)
GS 7b	2 (9%)	6 (43%)	7 (33%)	2 (33%)	2 (25%)	–	1 (8%)
GS 8	9 (39%)	2 (14%)	1 (5%)	3 (50%)	3 (38%)	3 (30%)	4 (33%)
GS 9–10	10 (43%)	5 (36%)	6 (29%)	–	3 (38%)	5 (50%)	4 (33%)
Unknown	–	–	1 (5%)	1 (17%)	–	–	2 (17%)
**pT Stage**							
pT2 (n)	4 (17%)	–	2 (10%)	–	2 (25%)	–	1 (8%)
pT3a (n)	11 (48%)	10 (71%)	10 (48%)	1 (17%)	3 (38%)	3 (30%)	2 (17%)
pT3b^a^ (n)	8 (35%)	4 (29%)	8 (38%)	4 (67%)	3 (38%)	7 (70%)	7 (58%)
Unknown	–	–	1 (5%)	1 (17%)	–	–	2 (17%)
**Lymph node dissection**							
Number of resected LN (mean (±STD))	20 (±7.2)	20.4 (±7.3)	19.7 (±6.7)	23.7 (±12.8)	19.8 (±6.3)	24.1 (±11.3)	21.4 (±6.2)
Number of positive LN (mean (±STD))	1.4 (±0.7)	1.1 (±0.3)	1.5 (±0.7)	3.7 (±1.4)	3 (±1.2)	3.5 (±1.8)	7.3 (±3.2)
Ratio positive LN/all LN (%)	8.3	4.6	8.7	17.1	15.7	18.4	37.0
**Follow-Up**							
PSA persistence (%)	50	20.8	42.9	50	50	70	91.7
1y BCRFS (%)	71.1	80	65.6	100	50	66.7	100^b^

*Ext* External Artery: *In*t Internal Artery: *Obt* Obturator fossa: *BCRFS* biochemical recurrence free survival: *GS* Gleason Score: *IQR* Interquartile range: *LN* lymph node

^a^No pT4 Stage found

^b^only 1 patient without PSA persistence, this one with no recurrence

To evaluate if the extended dissection with removal of the internal nodes has a therapeutic effect in addition to the diagnostic benefit, we evaluated the disease-free rate in men with positive nodes in this area; postoperative PSA persistence was observed in 61.0% (25/41) of patients with positive internal iliac nodes.

**Fig. 3:**
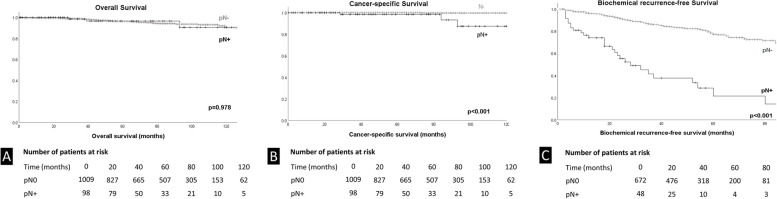
Kaplan-Meier curves for (**A**) overall survival (OS), (**B**) cancer-specific survival (CSS) and (**C**) biochemical recurrence-free survival (BCRFS) after robot-assisted laparoscopic radical prostatectomy stratified for pN0 and pN+. Patients not reaching a PSA nadir of < 0.1 ng/mL were not included in BCRFS analysis. *P* values were calculated using Log-Rank test

The median (IQR) follow-up time for patients alive was 59 months (IQR 28–85). Deaths of any cause and from PCa occurred in 46 patients (4.2%) and in 4 patients (0.4%), respectively. The estimated OS at 5 years was 96.5% for pN+ and pN- men, and at 7 years 91.5 and 94.3%. respectively (Fig. 3A, *p* = 0.98). The estimated CSS at 5 years was 98.5% for pN+ and 100% for pN- men, and at 7 years 93.3 and 99.7%, respectively (Fig. 3B, *p* < 0.01). There was no significant association between a certain N+ region (internal, external, obturator or multiple) and the CSS and OS rate (Supplementary Fig. 1).

In pN+ patients achieving a postoperative PSA nadir of < 0.1μg/ml (*n* = 47), the estimated 5-year BCRFS, CSS and OS were 17.3% (Fig. 3C), 100 and 100%, respectively. The median time to BCR was 21 months (IQR 8–34). In this subgroup, salvage EBRT, deferred ADT or a combination was applied in 12 (25.5%), 5 (10.6%) and 10 (21.3%) men, respectively (Table 3). In the univariate cox regression analysis, presence of LN metastasis was a predictor of shorter BCRFS (OR 6.936, 95% CI 4.63–10.38, *p* ≤ 0.001, Supplementary Table 2). Patients with 1, 2 or more than two positive LNs had an estimated median BCRFS of 28, 18 and 21 months.

**Table 3:** Postoperative therapy in nodal positive men

**A)**				
**Postoperative therapy**	**Nadir reached (*n = 47*)**	**Postoperative therapy**	**PSA persistence (*n = 50*)**	
Salvage EBRT	12 (25.5%)	Adjuvant EBRT	10 (20%)	
Deferred ADT	5 (10.6%)	Immediate ADT	18 (36%)	
Combination	10 (21.3%)	Combination	14 (28%)	
None	20 (42.6%)	Therapy declined	8 (16%)	
**B)**				
**Time to therapy**	**Nadir reached**	**PSA persistence**	**All pN+**	**p-value**
Median time (months) to EBRT (range)	13 (3-82)	7 (2-110)	9 (2-110)	0.261
Median time (months) to ADT (range)	21 (0-106)	4 (1-89)	5 (0-106)	0.062

A: Postoperative therapy in pN+ patients stratified for men reaching a postoperative PSA Nadir of < 0.1 ng/ml and PSA persistence

B: The average time in months between surgery and external beam radiotherapy (EBRT), androgen deprivation therapy (ADT) or a combination

In pN+ patients with postoperative PSA persistence (*n* = 50), adjuvant EBRT, immediate ADT or a combined approach was performed in 10 (20%), 18 (36%) and 14 (28%) men, respectively (Table 3). Eight patients (16%) declined any adjuvant therapy. In this cohort, the 5-year CSS and OS were at 97.2 and 93.5%, and at 7 years, respectively, remained at 97.2 and 93.5%.

The average time between surgery and EBRT, ADT or a combination are displayed in Table 3. We observed a shorter time to EBRT (7 vs. 13 months, *p* = 0.2) and ADT (4 vs. 21 months, *p* = 0.06) in men with PSA-persistence compared to men reaching non-detectable levels postoperatively. In pN+ patients achieving a postoperative PSA nadir of < 0.1μg/ml (*n* = 47), patients who received salvage radiotherapy alone or in combination with ADT as well as patients without salvage radiotherapy had a 5-year overall survival of 100% (*p* = 0.564).

In pN+ patients with biochemical persistence, the 5-year overall survival rate was 88.9% in patients without adjuvant radiotherapy, compared to 100% in patients with adjuvant radiotherapy alone or in combination with ADT (*p* = 0.639).

## Discussion

Earlier studies on region specific LN yields and metastatic patterns were based on open prostatectomy series since within laparoscopic procedures LNs are typically merged and placed in endobags before retrieval [16]. In this large series of RARC with ePLND LN packages of each region were retrieved through the access port and send in separately for pathological workup enabling an in-depth analysis of LN yields stratified for side and anatomical region. Based on this detailed pathological workup we could observe a significantly higher number of retrieved LNs on the right side compared to left. This superior yield translated also into a significantly higher number of positive LNs diagnosed on the right side. Notably, no prior investigation on robotic PLND during RARP has reported site-specific lymph node yield [20–24]. A physiological imbalance of pelvic LNs is rather unlikely, although this cannot be excluded due to lack of data from anatomical studies or open series [16, 25]. Only one study with a limited number of patients reported a tendency to a higher number of positive LNs on the right than on the left side [26] without presenting the total number on each side. We hypothesise that the technical set-up of the robotic approach is the most likely reason for differing LN yields; First, the typical localization of the grasping instrument on the left allows a decent access to the lymphatic tissue behind the iliac vessels and lateral to the obturator nerve on the right, while on the left the access is limited due to the working angle. Second, the fourth arm typically placed on the left can be used for an enhanced exposure of the internal LNs on the right through traction on the peritoneum. Third, the localisation of the sigma on the left may lead to a more cautious preparation of the internal LNs. Surgeons should be aware of this potential limitation of laparoscopy, although the clinical relevance remains unknown.

The benefits of ePLND must be weighed against potential drawbacks of the procedure. Intraoperative complications related to PLND are linked to the anatomical structures in the surgical field and include injuries to the pelvic vessels, ureter, and obturator nerve. Additionally, dissection of lymphatic tissue can lead to symptomatic lymphoceles, lymphoedema and thromboembolic events. Fortunately, available scientific evidence shows that ePLND during RARP is safe with low rates of intraoperative and postoperative complications [27]. Indeed, we could confirm this favorable outcome data on ePLND and did not observe any relevant injuries to the surrounding structures. Notably, we noticed a higher transfusion rate in the non-ePLND group. The dissection might have been omitted more often in men at increased risk for bleeding and RARPs without PLND performed earlier on the learning curve of surgeons. The rate of symptomatic lymphoceles and reoperations were low but higher than in the cohort without dissection and increased over time. A possible explanation for this development may be a more broad indication for RP and ePLND in regards to age, BMI, comorbidities and anticoagulants with gaining experience of the surgeons.

In an analysis of affected lymphatic sites we observed a lower rate of metastasis in the internal iliac region and a lower rate of men with exclusive involvement of these nodes (Fig. 2A/B) confirming the proposed lower rate of lymphatic drain from the prostate through this route [25]. To further investigate, if a specific metastatic pattern is a consequence of more advanced disease, we performed an in-depth analysis of LN metastasis based on region and correlated the results with oncological outcome. We did not find an association of involved regions with recurrence; notably, involvement of internal nodes was not associated with a more unfavorable oncological outcome. These findings underline that there is rather a heterogeneous lymph drain from the prostate resulting in various patterns of LN involvement in the pelvis than regional involvement in the pelvis based on disease stage or aggressiveness of the tumor.

There is still a lack of scientific evidence clarifying whether PLND is only a staging procedure or a therapeutic intervention [28]. In the present cohort the value of ePLND for accurate staging has to be questioned; more than half of the nodal positive men (50/97) had a postoperative PSA persistence, which would qualify them for early postoperative imaging with PSMA PET CT and adjuvant treatment irrespective of the nodal status. Therefore, a potential diagnostic benefit based on ePLND at the time of surgery was only achieved for 47/823 (5.7%) men with non-detectable PSA after RALP. However, since a deferred treatment after biochemical recurrence was the preferred strategy in these men, only the modality of follow-up might have been affected. In regard to the oncological benefit of ePLND in terms of tumor control, the value of ePLND also remains questionable. Among the 47 men with non-detectable PSA postoperatively most experienced an early recurrence resulting in ADT and/or EBRT (5y-BCRFS of 17.3%). From the overall cohort, only 20/823 (2.4%) had nodal positive disease with postoperative non-detectable PSA who could be spared from additional treatment during follow-up. The number of ePLNDs needed to perform to avoid ADT and/or EBRT in one patient was 41. However, if the achieved disease-free status of these very few men can be attributed to the ePLND, as postulated before [28], remains uncertain and such a question can only be answered accurately in a prospective randomized clinical trial.

Finally, we performed a subgroup analysis to assess the potential diagnostic and therapeutic value of the extended template for PLND observing that a low priority can be given to the internal nodes; only 15 men (1.8%) would have been falsely staged as nodal negative if the internal nodes had not been dissected. Metastatic tissue would have been left in situ in 42 (5.1%) of patients. Among these men, 25 (60%) experienced PSA persistence and most likely did not benefit oncologically from the dissection.

### Limitations

Our study has several limitations. It is a single-center retrospective study, and we cannot rule out that the site-specific surgical technique and pathological workup may have affected the reported LN numbers. The decision whether to perform PLND was not based on an established nomogram such as Briganti, but dependent on counseling of the treating urologist. A more stringent selection of patients would probably have led to a more favorable cost-benefit relation for ePLND, reducing the number of ePLND to be perfomed to spare a patient from ADT and/or EBRT. Further, during follow-up we recorded only the rate for symptomatic lymphoceles, reoperations, and transfusion rates but for no other complication. Data on metastasis-free survival, location of recurrence or time to castration-resistance was not available.

Finally, adjuvant treatment was not standardized and no data on dose and field for postoperative radiation therapy was available.

## Conclusion

In this cohort of men undergoing robotic ePLND a high LN yield could be achieved with a low complication rate. However, we observed an imbalance in more removed and positive LNs on the right side compared to left. The majority of patients with positive lymph nodes undergoes adjuvant treatment due to PSA persistence or early biochemical recurrence, indicating a possibly limited therapeutical value of ePLNDin already spread disease. Yet, these men demonstrated an excellent 7y OS and CSS.

### Supplementary Information

**Additional file 1:** **Supplementary Table 1. **Postoperative complications.

**Additional file 2: Supplementary Figure 1.** Kaplan-Meier curves for (A) overall survival (OS), (B) cancer-specific survival (CSS) and (C) biochemical recurrence-free survival (BCRFS) after robot-assisted laparoscopic radical prostatectomy stratified for patients with different positive LN regions. Patients not reaching a PSA nadir of <0.1 ng/mL were not included in BCRFS analysis. *P* values were calculated using Log-Rank test.

**Additional file 3:** **Supplementary Table 2.** A Univariate logistic regression analysis for prediction of postoperative PSA persistence. B Univariate Cox Regression Analysis for prediction of biochemical recurrence.

## Data Availability

The datasets during and/or analysed during the current study available from the corresponding author on reasonable request.
