# Supplementary material for: Outcomes of robot-assisted laparoscopic extended pelvic lymph node dissection for prostate Cancer
Source: BMC Urol. 2024 Jan 29;24:24. doi: 10.1186/s12894-024-01409-8 (PMC10823685; doi:10.1186/s12894-024-01409-8)
Supplement: Supplementary file 1 — Additional file 1: Supplementary Table 1. Postoperative complications. [file 12894_2024_1409_MOESM1_ESM.docx]

**Supplementary Table 1 Postoperative complications**

| Complications | ePLND (n=827) | no ePLND (n=280) | OR (95% CI) | p-value |
| --- | --- | --- | --- | --- |
| EC administration | 17 (2.1%) | 13 (4.7%) | 0.43 (0.21-0.90) | 0.024 |
| Lymphocele* | 19 (2.3%) | 0 (0%) | NA | 0.004 |
| Clavien Dindo Grade IIIa  Clavien Dindo Grade IIIb | 13 (1.6%)  27 (3.3%) | 1 (0.3%)  3 (1.1%) | 3.49 (1.24-9.84) | 0.012 |

Supplementary table 1: postoperative complications within 90 days postoperative of patients undergoing robot-assisted laparoscopic radical prostatectomy with and without ePLND.

*clinically relevant (symptoms or in need for intervention)

EC: erythrocyte concentrate, PLND: pelvic lymph node dissection, OR: odds ratio,
