# Supplementary figures and images for: Outcomes of robot-assisted laparoscopic extended pelvic lymph node dissection for prostate Cancer
Source: BMC Urol. 2024 Jan 29;24:24. doi: 10.1186/s12894-024-01409-8 (PMC10823685; doi:10.1186/s12894-024-01409-8)

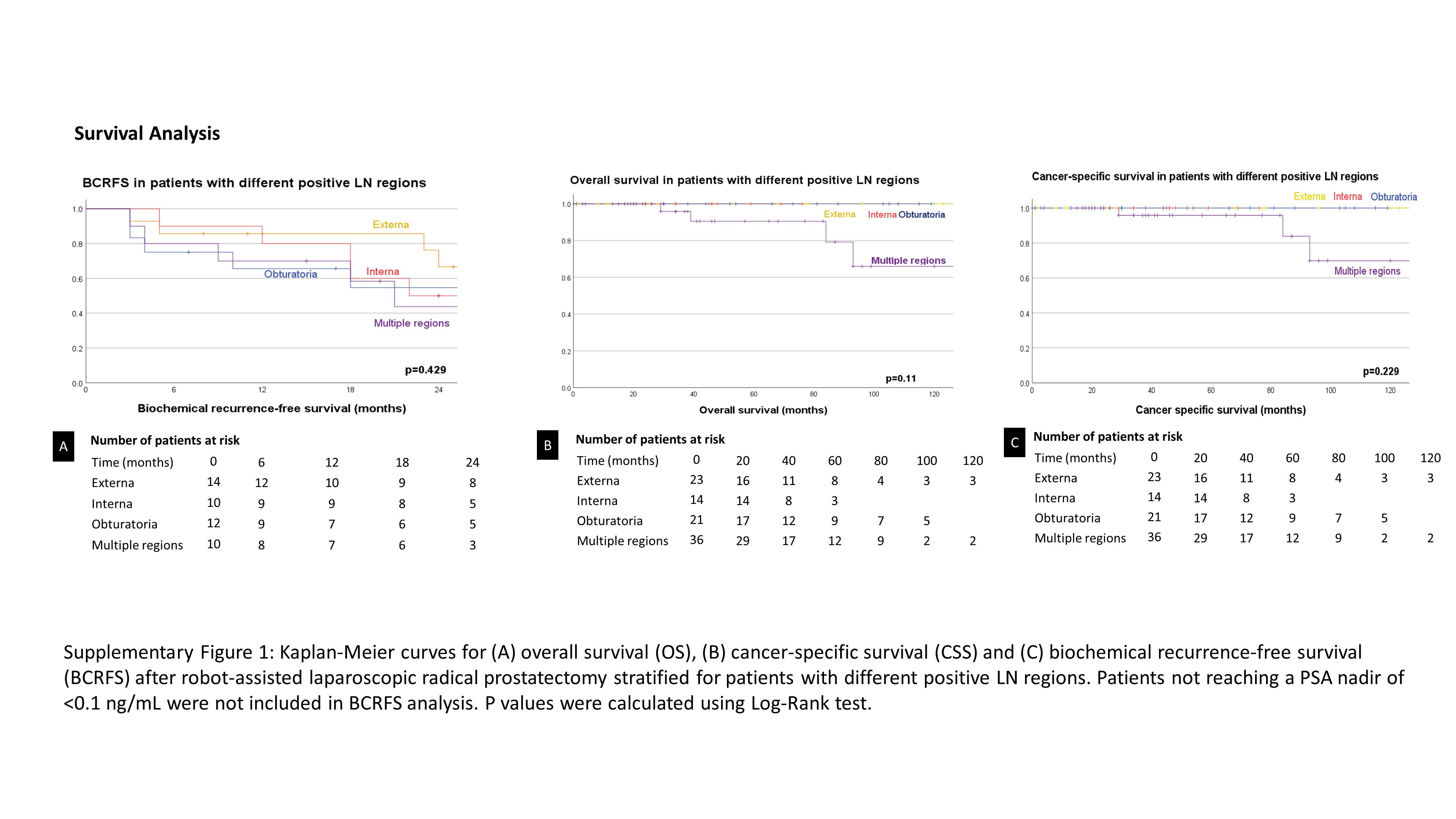

Supplement: Supplementary file 2 — Additional file 2: Supplementary Figure 1. Kaplan-Meier curves for (A) overall survival (OS), (B) cancer-specific survival (CSS) and (C) biochemical recurrence-free survival (BCRFS) after robot-assisted laparoscopic radical prostatectomy stratified for patients with different positive LN regions. Patients not reaching a PSA nadir of <0.1 ng/mL were not included in BCRFS analysis. P values were calculated using Log-Rank test. [file 12894_2024_1409_MOESM2_ESM.tif]
