# Supplementary material for: Outcomes of robot-assisted laparoscopic extended pelvic lymph node dissection for prostate Cancer
Source: BMC Urol. 2024 Jan 29;24:24. doi: 10.1186/s12894-024-01409-8 (PMC10823685; doi:10.1186/s12894-024-01409-8)
Supplement: Supplementary file 3 — Additional file 3: Supplementary Table 2. A Univariate logistic regression analysis for prediction of postoperative PSA persistence. B Univariate Cox Regression Analysis for prediction of biochemical recurrence. [file 12894_2024_1409_MOESM3_ESM.docx]

**Supplementary Table 2**

**Table 2A Univariate logistic regression analysis for prediction of postoperative PSA persistence**

|  | OR | 95% CI | p-value |
| --- | --- | --- | --- |
| Age (y) | 1.0 | 0.97-1.03 | 0.735 |
| PSA (ng/ml) | 1.1 | 1.06-1.10 | <0.001 |
| Tumor stage |  |  |  |
| pT2 | Reference | - | - |
| pT3a | 5.6 | 3.34-9.43 | <0.001 |
| pT3b | 17.2 | 9.81-30.25 | <0.001 |
| Positive surgical margin | 4.0 | 2.66-6.05 | <0.001 |
| No. of removed LNs* | 1.0 | 0.97-1.02 | 0.797 |
| No. of positive LNs* | 2.0 | 1.64-2.49 | <0.001 |
| pN+ | 17.2 | 10.64-27.7 | <0.001 |
| Gleason Score |  |  |  |
| 6 | Reference | - | - |
| 7a | 1.7 | 0.73-4.13 | 0.216 |
| 7b-10 | 8.0 | 3.36-19.24 | <0.001 |

*LN: lymph node, OR: Odds Ratio, y: year*.

* in patients undergoing pelvic lymph node dissection

**Table 2B Univariate Cox Regression Analysis for prediction of biochemical recurrence**

|  | OR | 95% CI | p-value |
| --- | --- | --- | --- |
| PSA (ng/ml) | 1.036 | 1.022-1.050 | <0.001 |
| Pathological stage |  |  |  |
| pT2 | Reference | - | - |
| pT3a | 4.423 | 3.185-6.144 | <0.001 |
| pT3b/pT4 | 8.032 | 5.323-12.121 | <0.001 |
| Positive surgical margin | 2.551 | 1.903-3.421 | <0.001 |
| No. of removed LN* | 1.000 | 0.983-1.018 | 0.991 |
| No. of positive LN* | 1.648 | 1.426-1.904 | <0.001 |
| pN+ | 6.936 | 4.634-10.380 | <0.001 |
| Gleason score |  |  |  |
| 5-6 | Reference | - | - |
| 7 | 3.034 | 1.624-5.671 | <0.001 |
| 8-10 | 9.470 | 4.991-17.969 | <0.001 |

*LN: lymph node,* *OR: Odds Ratio,*

* in patients undergoing pelvic lymph node dissection
